# *Getting to Implementation* for HIV Pre-Exposure Prophylaxis (GTI-PrEP): A data-driven approach to PrEP prescribing

**DOI:** 10.1186/s43058-025-00749-2

**Published:** 2025-06-02

**Authors:** Shari S. Rogal, Brittney Neely, Monica Merante, Karen Slazinski, Lorenzo McFarland, Christina Brodkorb, Jada Cooper, Carolyn Lamorte, Sandra Gibson, Emily Comstock, Jamie Morano, Marissa Maier, Lauren A. Beste, Karine Rozenberg, Maggie Chartier, Matthew J. Chinman, David Ross, Vera Yakovchenko

**Affiliations:** 1 Center for Health Equity Research and Promotion, VA Pittsburgh Healthcare System, Pittsburgh, PA USA; 2 Division of Gastroenterology, Hepatology, and Nutrition, University of Pittsburgh, Pittsburgh, PA USA; 3 Department of Surgery, University of Pittsburgh, Pittsburgh, PA USA; 4 Infectious Diseases Section, Orlando VA Medical Center, Orlando, FL USA; 5 Office of Specialty Care Services, Department of Veterans Affairs, HIV, Hepatitis, and Related Conditions, Washington, DC USA; 6 Gulf Coast Veterans Health Care System, Biloxi, MS USA; 7 Infectious Diseases Section, Baltimore VA Medical Center, Baltimore, MD USA; 8 Department of Medicine, Infectious Disease Section, Tampa VA Medical Center/James A. Haley Veterans’ Hospital, Tampa, FL USA; 9 Infectious Diseases Section, VA Portland Health Care System, Portland, OR USA; 10 Department of Medicine, Division of Infectious Diseases, Oregon Health & Science University, Portland, OR USA; 11 General Medicine Service, Puget Sound VA Healthcare System, Seattle, WA USA; 12 Division of General Internal Medicine, University of Washington, Seattle, WA USA; 13 VA Great Lakes Health Care System, VISN 12 Pharmacy Benefits Management, Westchester, IL USA; 14 Office of Specialty Care Services, Veterans Health Administration, Washington, DC USA; 15 RAND Corporation, Pittsburgh, PA USA; 16 Moderna, Inc, Cambridge, MA USA

**Keywords:** Implementation, Strategies, Healthcare delivery, HIV/AIDS, Pre-exposure prophylaxis, Prevention

## Abstract

**Background:**

Pre-Exposure Prophylaxis (PrEP) dramatically reduces the likelihood of acquiring human immunodeficiency virus (HIV), yet it remains under-prescribed, particularly for people in communities with high HIV incidence. While implementation science and health services researchers aim to address disparities in care, few interventions have proven effective in doing so. We aimed to identify implementation strategies associated with higher PrEP prescribing rates and pilot test a tailored intervention as a proof-of-concept in a single Veterans Health Administration (VA) facility.

**Methods:**

VA clinicians were surveyed using an instrument derived from the Evidence-based Recommendations for Implementing Change taxonomy to assess the use of various strategies for PrEP in fiscal years 2019–2021. Correlational analyses identified the strategies associated with the frequency of PrEP prescribing and semi-structured interviews with personnel from 11 VA medical facilities with high PrEP prescribing refined and manualized these strategies into the *Getting to Implementation* (GTI)-PrEP playbook. The playbook was subsequently pilot tested in a VA facility with high new HIV diagnosis rates and low PrEP prescribing rates.

**Results:**

The clinician survey collected 157 responses from 95 unique VA facilities on implementation strategy use. Analysis identified eight strategies significantly associated with PrEP prescribing, including: networking, clinician education, clinical support tools, dashboard utilization, telehealth, pharmacist involvement, direct patient engagement, and enhanced sexual health history taking. In the pilot study, the site completed the GTI-PrEP Playbook with high fidelity and newly implemented seven of the eight strategies, achieving a 363% increase in PrEP prescribing rates among Black Veterans over the one-year period.

**Conclusions:**

This multi-year national evaluation identified a core subset of effective implementation strategies for increasing PrEP prescribing. The process of empirically specifying these strategies and pilot testing them through the GTI-PrEP playbook demonstrates a promising, data-driven approach to improve PrEP prescribing rates and reduce racial disparities in HIV prevention.

**Supplementary Information:**

The online version contains supplementary material available at 10.1186/s43058-025-00749-2.

Contributions to the literature •Uncertainty remains about whether implementation strategies identified through observational data can be “prescribed” to improve care delivery and reduce inequities in diverse settings.

•We evaluated strategy use for Pre-Exposure Prophylaxis (PrEP) and adapted a manualized approach—Getting to Implementation—to specifically enhance PrEP prescribing in the Veterans Health Administration.

•Pilot findings offer preliminary evidence for broader healthcare delivery interventions that a manualized toolkit with prescribed core implementation strategies can effectively improve PrEP prescribing.

## Background

In the United States (US), approximately 1.2 million people are living with human immunodeficiency virus (HIV), with more than 31,800 new diagnoses annually [1, 2]. Pre-Exposure Prophylaxis (PrEP) is a highly effective evidence-based practice that can reduce HIV acquisition by over 99% among people exposed to HIV through sexual contact [2, 3]. Since its release in 2012, interventions continue to support PrEP implementation as it nears its 17-year evidence-to-practice gap [4–6]. Recognizing its efficacy, the United States Preventive Services Task Force, Centers for Disease Control and Prevention (CDC), and Veterans Health Administration (VA) recommend clinicians offer PrEP to persons who are more likely to acquire HIV infection, with the hopes of reaching Ending the HIV Epidemic Initiative goals to reduce new HIV infections by 90% [2, 7, 8].

Despite these consensus recommendations, PrEP prescribing remains low, with significant disparities across geographic and demographic groups [9–11]. The Southern US, for instance, accounts for approximately half of all new infections, but only 39% of PrEP users, indicating a substantial implementation gap. Moreover, Black or African Americans are the least likely to receive PrEP, representing only 14% of PrEP users, yet accounting for 42% of all new HIV diagnoses [12]. This inequitable and low adoption of PrEP represents an evidence-to-practice gap requiring attention.

As the largest integrated healthcare system in the US, the VA has sought to address these gaps by increasing PrEP prescribing among eligible Veterans. In February 2020, the leadership team of the VA HIV, Hepatitis, and Related Conditions Program (HHRC) launched the VA National HIV Affinity Group program. Modeled after the joint effort by the Centers for Medicaid and Medicare Services, CDC, and Health Resources and Services Administration [13], this VA affinity group initiative aims to promote and support high quality HIV prevention and care access VA-wide. An evaluation was conducted to understand both the barriers to quality improvement and the successful strategies associated with improved implementation.

A persistent challenge in implementation science is selecting and tailoring strategies to setting-specific barriers [14, 15]. The Expert Recommendations for Implementing Change (ERIC) taxonomy of 73 strategies provides a standard approach to catalog and select bundles of strategies associated with higher performance on various implementation measures [16–18]. However, an open question in implementation research is whether strategy bundles identified through observational data can be “prescribed” to improve implementation in different settings [19–21]. Additionally, it remains unclear how and to what extent implementation strategies can address systemic inequities and ameliorate disparities in healthcare delivery [22, 23].

Implementation strategies work best when they are selected and tailored to the needs of the implementation setting, yet it can be challenging to practically do so using an engaged approach on a clinically meaningful timeline [24]. Getting to Outcomes (GTO) is a structured 10-step approach that allows organizations to plan, implement, and evaluate *interventions* across a range of issues including substance use, housing instability, and teen pregnancy [25–27]. Building on the effectiveness of GTO, our team adapted this approach to support hospitals in selecting implementation strategies (rather than interventions) [21, 28]. The resulting Getting to Implementation (GTI) offers a stepwise approach to help hospitals select, tailor, and evaluate implementation strategies [21].

For the present study, we aimed to 1) identify implementation strategies associated with high PrEP prescribing rates across VA facilities nationwide, 2) specify and manualize these core strategies into Getting to Implementation for HIV PrEP (GTI-PrEP), and 3) pilot test GTI-PrEP to increase PrEP prescribing in one VA facility.

## Methods

### Overview of study design and approval

**Fig. 1:**
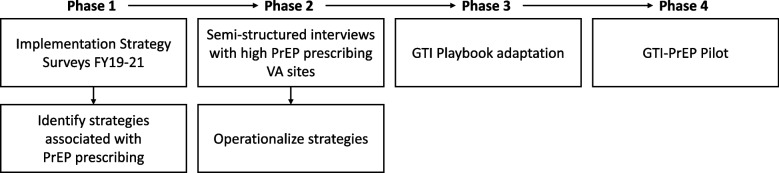
Explanatory sequential mixed methods

This was an explanatory sequential mixed methods study conducted in four phases (Fig. 1). Phase 1 involved quantitative survey of HIV/PrEP clinicians to assess prescribing rates, associated strategies, and identify sites for the pilot phase. Phase 2 involved conducting semi-structured interviews with the highest PrEP prescribing sites to enrich and contextualize Phase 1 quantitative findings. Phase 3 focused on adapting the GTI Playbook to include these strategies, making GTI-PrEP. Finally, Phase 4 involved pilot testing GTI-PrEP in a representative VA facility with baseline low PrEP prescribing and opportunities to improve equity of PrEP prescribing (i.e., more equal rates of PrEP prescribing across patient groups). This quality improvement project was developed by HHRC within the VA Office of Specialty Care Services and VA’s Office of Health Equity to help VA facilities improve utilization of PrEP. Per regulations outlined in VA Program Guide 1200.21 [29], this project was deemed a non-research operations activity. Participation in the evaluation was voluntary. Standards for Quality Improvement Reporting Excellence (SQUIRE) were applied and are reported upon in Additional File 1.

### Phase 1: Clinician Survey

#### Survey development

We conducted annual implementation strategy surveys for HIV prevention and care improvement in fiscal years (FY) 2019–2021 with VA clinicians. We worked closely with HHRC leadership and subject matter experts in the infectious disease (ID), medical, and pharmacy fields to develop a comprehensive survey of tailored ERIC implementation strategies. In the first year, ERIC strategies were contextualized to the PrEP clinical area and the VA setting or added/removed based on recommendations from operational partners resulting in 58 of the original 73 ERIC strategies being retained and 14 strategies being added. In the second year, strategies had minor modifications (one strategy was cut and three were added). In the third year, based on strategy use data from prior years and through continued consensus discussion with partners, 39 strategies were cut (19 strategies were cut/subsumed due to partners’ priority shift, seven strategies were combined due to strong ties with pre-existing strategies, six strategies were cut due to low use) and four were added (two of these strategies were created by splitting a strategy from the previous year into two separate questions). The excluded strategies focused on immutable financial and policy changes, while the added split strategies specified modes of delivery. The final 39 survey items evaluated 33 of the original 73 ERIC strategies (Table 1).

**Table 1:** Use of PrEP Implementation Strategies Across Three Years

Strategy	FY19% (*n* = 74)	FY20% (*n* = 37)	FY21% (*n* = 46)
**• Change physical structure (e.g., expand clinic space)**	14%	16%	**4%**
• Change equipment, medical supplies (e.g., get telehealth equipment, add rapid point of care testing)	30%	38%	19%
**• Enhance medical record systems (e.g., use clinical reminders, develop note templates, CPRS/Cerner order sets)**	43%	30%	**30%**
• Extend care into the community (e.g., with the MISSION Act)	9%	3%	2%
• Pull Veterans back into VA care from community care	-	-	19%
• Change local policy (e.g., allow access to needle exchange)	11%	3%	4%
• Hire new staff (e.g., RN/APRN, SW, clinical care navigators, PharmD, MSAs, schedulers)	16%	**11%**	15%
• Receive funding from new sources (e.g., apply for VA or non-VA grants, industry funding)	9%	5%	4%
• Collaborate with non-VA organizations to reduce financial burden (e.g., public health labs or outside commercial labs to reduce costs of testing)	7%	5%	0%
**• Create new clinical teams (e.g., develop an ID-PACT, add pharmacist to the team)**	**26%**	16%	**9%**
**• Transition from ID-PACT model to a co-managed PC and ID care model**	-	-	**4%**
• Revise staff roles or adjust existing clinical teams to encourage operating at top of license	30%	11%	13%
**• Develop resource service agreements (e.g., with another service line such as lab for self-collection)**	7%	0%	**11%**
• Tailor patient care (i.e., use special approaches to reach specific populations such as patients with sexual health indicators or patients in SUD clinic)	31%	**27%**	28%
• Use a clinical video telehealth (CVT) program	36%	**35%**	45%
• Use telephone encounters or clinics (e.g., for follow-up visits)	-	**57%**	81%
• Conduct educational or in-service trainings for primary care providers in HIV care and HIV prevention	-	-	43%
• Conduct educational or in-service trainings for ID prescribers and clinicians (MD/DO, PA/NP, SW, RN, PharmD, APRN) providing HIV care and HIV prevention (NOT primary care providers)	-	-	23%
• Join a national group such as a learning collaborative or work group for sharing best practices (e.g., HIV Prevention and Care Affinity Group)	24%	14%	47%
• Provide VA clinician-to-clinician consultation via shadowing and expert supervision (e.g., VA-ECHO)	73%	**43%**	4%
• Use external clinician training (e.g., professional organizations like AETC, AAHIVM, CDC)	32%	14%	26%
• Require VA TMS PrEP module as qualification for primary care providers to prescribe PrEP	**26%**	16%	6%
• Participate in national VA HIV/PrEP update calls (e.g., HIV Lead Clinician call, HIV TAG call)	62%	**43%**	55%
• Provide training or consultation to address stigma and discrimination (e.g., how to provide a welcoming environment to LGBTQ Veterans, Veterans of color, integrate anti-racism principles)	27%	16%	11%
• Provide training to improve taking a sexual history or screening for sexual risk	28%	22%	15%
**• Communicate with local facility VA leaders to gain support for HIV quality improvement**	14%	5%	**13%**
• Develop a local facility group/team to learn together or to address challenges/barriers	11%	3%	9%
**• Share the knowledge gained from quality improvement efforts with other facilities or CBOCs**	**14%**	5%	**13%**
• Have a local champion promote HIV care or PrEP (can be different from HIV Lead Clinician)	**18%**	11%	40%
• Seek guidance and support from implementation experts (e.g., academic detailing, coaches, technical assistants, quality improvement staff)	12%	3%	6%
**• Partner with an academic medical center for the purposes of sharing training opportunities and developing research skills**	20%	5%	**21%**
• Prepare for widescale implementation by learning from early adopters, other facilities, and/or pilot studies	16%	3%	6%
**• Assess how ready your site is to make quality improvement changes**	18%	8%	**13%**
• Develop a written plan of goals, and strategies to accomplish goals (e.g., develop team charter)	**14%**	**16%**	6%
• Inform clinicians/administrators of quality improvement results using clinical performance data	**14%**	11%	11%
**• Develop or use quality monitoring tools (e.g., PrEP dashboard, STI dashboard, HIV Cube)**	**12%**	**14%**	**55%**
**• Provide patients the option to self-collect for STIs (e.g., GC/CT)**	-	**24%**	**34%**
• Engage with veterans and their families to promote retention in care	61%	24%	34%
**• Create or use print/social media to reach patients**	22%	5%	**17%**

Note: Bold denotes positive association with PrEP prescribing,—denotes strategy item was not asked in the year

#### Survey recruitment

HHRC leadership announced the annual survey on national calls and emailed this voluntary survey each year to the VA-wide HIV Clinicians listserv, the HIV Technical Advisory Group, and VA HHRC Affinity Group members. The HHRC team sent two reminder emails to boost survey completion, and recipients were encouraged to forward the survey to others (i.e., snowball sampling) with knowledge about HIV prevention and care in their facility. Following VA protocol, clinicians were not financially incentivized to complete the survey.

#### Survey analysis

Survey analyses were conducted using R and RStudio to identify a core subset of strategies consistently linked to PrEP prescribing in years FY19-21. We evaluated data from the VA Corporate Data Warehouse (CDW) and calculated PrEP rates as [number of Veterans receiving PrEP]/[number of Veterans at each VA facility per 100,000]. Non-parametric statistical tests were used to assess the association between strategy use and PrEP prescribing. Strategies identified as significant were selected for further exploration in interviews after discussion with operational partners.

### Phase 2: Specifying Strategies

A semi-structured interview guide was created to capture the strategy specifications in high-performing VA facilities, following Proctor et al. guidance [30] about specification (e.g., define what, why/justify, who, when/dose, goal/outcome, barriers, facilitators). HHRC leadership reviewed the interview guide and provided edits and feedback. We identified 17 VA facilities in the highest quartile of guideline-concordant PrEP prescribing at the close of FY21 (September 2021) to ensure diversity in terms of size, location, and rurality. Key respondents (e.g., known as clinical champions) from these 17 facilities received an email inviting them to participate in a 45-min Microsoft Teams interview conducted in October 2021-February 2022. With permission of participants, interviews were audio recorded and transcribed. Two coders (MMM and BN) extracted detailed strategy specifications from each facility, then integrated across facilities to establish a core operationalization for each strategy to be verified with the senior author (SSR).

### Phase 3: Developing GTI-PrEP

GTI was initially an adaptation of RAND’s GTO [25, 26, 31, 32]. Details on this adaptation process to account for the VA context and implementation strategy content area have been previously described [28]. Next, GTI was tailored to incorporate strategy operationalizations identified and found to be effective for PrEP prescribing in Phase 1 and 2. The tailoring process was iterative, involving collaborative review and decision-making by GTO developers, HHRC leadership, clinical experts, and GTI facilitators/evaluators.

### Phase 4: GTI-PrEP pilot

#### Training facilitators on GTI-PrEP

GTI facilitation was to be led by a two-person team, including a clinical expert facilitator and evaluation facilitator with social work, implementation science and evaluation experience. GTI-PrEP developers and GTI evaluation facilitators trained five HHRC partners intended to be clinical expert facilitators in May 2022. Training included interactive explanation of the theory and development of GTO/GTI, the steps, and guided role plays of the worksheets, as well as best practices in facilitation.

#### Implementation

The pilot site was chosen based on being located in an Ending the HIV Epidemic priority jurisdiction (i.e., a CDC-defined area with high numbers of new HIV diagnoses), baseline HIV prevalence, low overall PrEP prescribing, and racial disparities in prescribing that disfavored Black Veterans [33]. The GTI team sent an initial outreach email to the pilot VA facility leadership in July 2022 and held an introductory meeting with facility staff and leadership. The facility leadership signed the participation agreement in September 2022 and identified a team of clinicians who would work with the facilitation team from September 2022 to September 2023, with site graduation in October 2023.

#### Evaluation

The pilot included five evaluation elements: 1) reach and equity of reach of PrEP prescribing over time, 2) GTI process and facilitation implementation (via a time-motion tracking form) 3) pre-GTI barriers to and facilitators of implementation (defined using a CFIR-based survey), 4) use and fidelity of effective strategies from the GTI manual to address barriers (tracked through meeting notes and observation and coded as medium or high), and 5) pilot site team feedback.

First, PrEP prescribing data were obtained annually by race/ethnicity and sex from CDW, and rates of change were calculated for each subgroup. Second, the GTI facilitator used a standard tracking sheet to document each facilitation event, and data were summarized descriptively [34]. Third, site team members completed a survey of CFIR-defined determinants of implementation. This survey offered each item phrased as a facilitator to implementation, where respondents could rank the extent to which the item served as a facilitator (strongly agree) or barrier (strongly disagree). Fourth, the GTI facilitator tracked and described which strategies the pilot site attempted during GTI. Fifth, qualitative data was collected throughout GTI via informal discussions with the facilitation team and more formally through a post-GTI semi-structured qualitative interview, then analyzed using a matrix synthesis analytic approach [35].

## Results

### Phase 1: Strategy survey

There were 157 responses from 95 unique VA facilities received across three years, including from 74 VA facilities in FY 2019, 37 in 2020, and 46 in 2021. Respondents included MDs (61%), APRNs (18%), clinical pharmacists (11%) and nurses (10%). Overall trends in the strategies used and strategy significance are shown in Table 1. Sites used a median of 12, 12, and 6 strategies for PrEP improvement in FY19-21, respectively. About a quarter (24–28%) of strategies were significantly associated with PrEP prescribing in each year. For example, “Develop and use quality monitoring tool” was significantly associated with PrEP prescribing in each year, while “Identify and prepare champions” was only significant in the first year and “Create or use print/social media to reach patients” was only significant in year three. Consensus discussions in Phase 1 resulted in a selection of eight strategies for operationalization in Phase 2, manualization in Phase 3, and inclusion in Phase 4 GTI-PrEP piloting.

### Phase 2: Specifying core strategies

Of the 17 sites invited to participate in an interview to operationalize strategies, 11 (65%) completed interviews. A total of 36 participants attended these interviews, with one to six per interview. These participants included physicians (*n* = 14), pharmacists (*n* = 12), nurses (*n* = 5), nurse practitioners (*n* = 2), a psychologist (*n* = 1), a physician assistant (*n* = 1), and a clinical program/benefits manager (*n* = 1).

Interviews were coded according to Proctor et al. specifications [30] to offer subsequent users information about how each strategy was conducted in other VA facilities, including who delivered the strategy and to whom, the forms that the strategy took, justification for the strategy, and resources to practically apply the strategy (e.g., sample templates). Table 2 illustrates the eight core strategies identified through surveys and operationalized from these interviews.

**Table 2:** Descriptions of strategies from high-performing VA facilities and descriptions of their application during GTI-PrEP

PrEP Core Strategy	High-Performer Quote (used to develop guide)	Application in Pilot Site (high or medium fidelity)
Network with VA and Community (1)	“I find those groups to just help me answer questions just like this with regards to like, “What is everyone else doing?” and listening to people discuss different barriers and how they overcome them, and it makes me feel I have a support group of peers who are also working through the same processes as we are. So, I think it sort of keeps me within the norm, and then also challenges me in certain ways…” (Site 11)	• Attended and presented on national calls with other clinicians and national leadership • Connected with local health department • (high fidelity)
Educate Clinicians and Staff (2)	“We met with different groups [from primary care] and it was very informal. It was like 3 or 4 people with me. We were sitting in an office talking and really answering every single question, going over everything, even going in the computer to guide them through how to do the order, how to put the medication, asking them about the level of comfort, decreasing a little bit about the anxiety.” (Site 8)	• Met with chief nurse • Sent educational email blasts • Brief discussions or “elevator chats” with providers and staff • Formal training presentations of baseline data, clinical reminders and tools, and sexual health history taking • (medium fidelity)
Use Clinical Support Tools (3)	“We've actually implemented the national HIV PrEP reminder. I'm not sure if any primary care providers are filling out those reminders…We tried to educate [primary care providers] a little bit when we first initiated it.” (Site 6)	• Installed and applied standard order sets, sexual history note template, and PrEP note templates, Sexual Orientation Clinical Reminder, and the PrEP lab order set • (high fidelity)
Use Patient Tracking Tools (Dashboards) (4)	“[The dashboard] would prompt me to do a chart review of the patient and then see if that's a good candidate for PrEP, and then if they did, I would put in a chart note that says, ‘This person’s got all these risk factors. They seem like a great candidate for PrEP.’ And then tag the PCP to see if they would consider referring them to the PrEP clinic.” (Site 5)	• Used National VA STI and PrEP dashboard weekly to identify possible HIV PrEP candidates to discuss with clinical team • Tracked positive STI/HIV results to offer social work and community referrals • (high fidelity)
Expand Access Through Telehealth (5)	“From the patient’s perspective, I've noticed really, significantly greater compliance with appointments since we've gone to VVC [telehealth]. They're more comfortable. Getting a sexual history is a very, very personal type of interviewing questions. So, the fact that they can be at home or in their car or wherever they feel comfortable, seems to work very, very well. And I’m sure you can tell as well, our no-show rate, and not that it was high before, but I feel it's extremely low now that we have the VVC option.” (Site 10)	• This strategy was being used in the VA facility prior to GTI and continued to be applied. During GTI, the LPN took novel steps to connect with the local County Health Department and obtain condoms/pamphlets for patient distribution • (medium fidelity)
Update the Role of Pharmacists (6)	“The importance of [pharmacists] cannot be understated. Just because of their presence in the clinic, their availability, their connection, and their relationship with all the different clinic providers. It's so important, and they do such a great job and I think the patients really appreciate having more regular access, having the phone access being able to just drop by the lab whenever it's convenient for them, get their labs drawn, do their swabs, do their urine, and then talk to somebody on the phone, get their medication in the mail. And so, we really have worked to make it as easy as possible for them and because of that we have really good retention in care, and I think a lot of that is because of our pharmacists.” (Site 7)	• Pharmacists started conducting medication use evaluation and placed PrEP referrals • Team leads met with pharmacy team to ensure communication and collaboration concerning order sets and medication management • (medium fidelity)
Engage Veterans Directly (7)	“We would either send a letter or she [the LPN] would send them a secure message through MyHealtheVet. So, we really wanted to empower the patient into knowing about pre-exposure prophylaxis and, if they’re interested, speaking with their primary care, or just reaching out to us directly so we could start the process.” (Site 10)	• After dashboard review, team members notified staff to encourage Veteran outreach or work with ID clinicians to contact patients directly to put in return to clinic orders to ensure needed labwork/medication refills for Veterans already receiving PrEP • (medium fidelity)
Promote Sexual History Taking (8)	“We gave Grand Rounds, [on] ‘How to ask sexually related questions.’ And we were teaching them how to ask, how to be very comfortable, how to approach open questions, not to be afraid of asking. The sexual history is not all about if you’re sexually active or not, but the whole nine yards and how to ask it.” (Site 8)	• Team members added LGBTQ + inclusive decoration and advertisements, encouraged clinicians to use the Sexual Health Assessment and Sexual Orientation Clinical Reminder, and to talk with HIV + Veterans about PrEP for their partners • (high fidelity)

### Phase 3: GTI-PrEP Playbook

Modifications to the GTI playbook were primarily content changes [36], including orienting to focus on equitable PrEP prescribing. Updates were made to each of the GTI steps and tools, including the Consolidated Framework for Implementation Research (CFIR) barrier and facilitator assessment tool language [37]. The resulting GTI-PrEP playbook comprised seven steps: 1) Build a team and identify current processes (i.e., how a Veteran receives a PrEP prescription); 2) Establish goals (i.e., equitable PrEP prescribing across racial groups, increase HIV testing); 3) Assess and prioritize strengths and barriers (e.g., identify which resources and facilitators are present, as well as identify challenges); 4) Choose solutions to increase the adoption or maintenance of PrEP prescribing (i.e., implementation strategies from Phase 1 and 2); 5) Plan and adapt solutions, tailoring the chosen strategies to a given facility and team; 6) Implement the work and evaluate how things go and what can be done better; 7) Sustain and look ahead (e.g., keep the efforts and momentum going long-term and beyond GTI-PrEP). A list of resources such as patient materials, clinical note templates, and information for new HIV clinicians was also added as an appendix.

### Phase 4: Pilot study

#### PrEP Outcomes

In the pilot site, new PrEP prescribing rates increased from 3.8/100,000 to 17.6/100,000 during the intervention year, representing a 363% increase. In contrast, the national rate increased from 73.0/100,000 to 87.6/100,000, reflecting a 20% increase. At the pilot site, PrEP prescribing rates also had significant growth across different demographic groups. Black and Hispanic Veterans experienced the highest increases, 146% and 179% respectively. Female Veterans saw a 92% increase in PrEP prescribing at the pilot site compared to a 32% increase nationally. National prescribing rates remained relatively stable for these subgroups, generally ranging between 20 and 32%, with the rates among male Veterans showing the least variation.

#### GTI process and facilitation implementation

Facilitators met with the pilot site team every two weeks, for a total of 14.5 h of prescheduled meetings and an additional 67.9 h of facilitation (ad hoc meetings, emails, messages, meeting preparation). The pilot site team included 29 members including a leadership-designated champion. The team members were from ID, GI, Women’s Health, Telehealth, Mental Health, Behavioral Health, Pharmacy, Primary Care, LGBTQ + Program, Social Work, Informatics, Clinical Applications, and Specialty Care. Meeting attendance ranged from three to 12, with an average of eight team members per call. Calls were structured around the GTI stepwise process and associated tools but purposefully kept flexible to allow the site team to address other issues as needed. GTI Steps focusing on strategies (Step 4, Step 6, and Step 7) received the most attention in terms of facilitation effort, as the pilot site team learned about, tailored, selected, and applied strategies.

#### Barriers and facilitators to implementation

For GTI Step 3, a pre-implementation baseline survey based on CFIR [37] identified two primary barriers, including 1) clinicians not having the time to complete necessary steps associated with PrEP prescribing and patient monitoring (domain: inner setting, construct: available resources), and 2) Not having an evaluation plan for assessing PrEP prescribing (domain: process, construct: reflecting and evaluating). In a subsequent pre-implementation interview with clinicians, additional barriers were uncovered, including competing primary care priorities, clinician and patient stigma surrounding HIV and PrEP, perceptions of patients’ low medication adherence, and large catchment area. Strengths were also noted during the interview, including leadership support, site team composition, site team commitment to PrEP improvement, ability to adapt current processes, and Veteran interest in PrEP.

#### Strategy selection

The GTI site was offered eight core strategies for selection identified in Phase 1 and operationalized in Phase 2. At the outset of GTI, the pilot site was implementing one (telehealth) of the eight strategies. The remaining seven strategies were selected and implemented by the site during GTI, and their application is described in Table 2. Four strategies were conducted with high fidelity to the prescribed operationalizations (networking with VA and community, promoting sexual history taking, using clinical support tools, and using patient tracking tools and dashboards) and three with medium fidelity (educating clinicians and staff, engaging veterans directly, and updating the role of pharmacists).

#### Qualitative Feedback

Site team members reported high satisfaction with GTI and how it made them feel “accountable” (NP) to pursue improvements in PrEP prescribing. One team member emphasized its effectiveness: “GTI does work. I will say if it wasn't for the GTI, the [PrEP] program wouldn't be running right now” (RN). The stepwise approach of GTI was particularly appreciated, as one team member noted: “So it really, really did help doing this in steps…That was my favorite part because it wasn't overwhelming. And it was work, but it wasn't overwhelming” (NP). The GTI facilitator played a key role in gauging site team readiness to advance through the GTI steps, despite occasional hesitancy from the site on their own perceived readiness to proceed.

A notable strength of GTI was its focus on early team formation. One participant reflected: “I would say that we didn't have a clue who we needed on the team” (RN), although another team member felt that some critical roles, such as the social worker and clinical applications manager, should have been identified “at the very, very beginning” (NP). Meeting coordination and busy clinician schedules required staff to “get creative” (NP) with finding time for GTI and the strategies. This was the first large-scale team effort to improve PrEP prescribing, which brought forth various team dynamics, including a lack of support between service lines and from leadership, thereby, at times, hindering progress and decision making. Staff turnover, specifically the loss of the clinician responsible for dashboard reviews, prevented continuous use of this core strategy.

Another consideration for staffing was related to stigma. One site member suggested that age, rather than sexual orientation or other patient-provider demographic concordance factors, might be more important relatable factors between as “it's necessary to have a younger person for them to talk to because I think they related a little bit more or feel like they relate instead of going to somebody that's not.” (RN). This perspective highlights the importance of considering various factors in patient-provider interactions, particularly in sensitive harm reduction conversations.

## Discussion

Implementation science offers strategies to address contextual barriers and leverage contextual facilitators [24, 30, 38, 39]. However, systematically prescribing strategies and matching them to barriers remains largely based on theory or expert opinion [24, 40]. This study aimed to advance this nascent area by identifying, specifying, and prescribing data-defined implementation strategies to address racial disparities in PrEP prescribing. GTI at the pilot site was successful, demonstrating promising results among Black, Hispanic, and female Veterans and providing a pragmatic method for applying implementation science principles to impact change.

The ERIC implementation strategy taxonomy provides content-agnostic definitions of 73 strategies to track implementation activities. However, ERIC strategy surveys must be tailored to the evidence-based practice (content), context, and language understood by the intended respondents. Over three consecutive years of data collection on HIV prevention and care in the VA, this study iteratively modified ERIC surveys to be responsive to partner interests in specific operationalizations of strategies. Although designed to be conceptually distinct, we modified  ERIC strategies at multiple levels to improve their relevance and clarity. Surface-level changes, such as minor wording adjustments and minimizing jargon were made, along with deep-level changes, including definition rephrasing, specifying strategy dimensions and mode of delivery, splitting/disaggregating, highlighting a separate step in a sequence, and combining strategies [41]. New strategies were created when existing ERIC strategies did not fully capture partner-identified needs, such as addressing stigma and promoting sexual history taking, which were not explicitly included in the original ERIC taxonomy. These modifications underscore the challenge of identifying a universal set of strategies applicable across contexts. By standardizing this tailoring approach of ERIC strategy surveys, this study both underscores the importance of engaging partners in implementation strategy survey development and adds to the growing knowledge about how to collect such data.

While the core strategies identified in this study to improve PrEP prescribing are not necessarily new, their systematic packaging and application through GTI-PrEP represent an innovation approach with promising results. The identified strategies align with prior findings, such as the dashboard strategy [42]. Combining direct patient outreach with the dashboard’s proactive, population health management approach to patient identification can potentially mitigate disparities that arise when PrEP prescribing is limited to patient-initiated requests. The additional core strategies addressed other gaps in the PrEP workflow, including provider preparation (education, networking), role delineation (expanding telehealth and the role pharmacists), documentation of efforts, and patient engagement (direct outreach, sexual history taking).

However, one of the key strengths of GTI-PrEP was its facilitated approach, which created a culture of accountability within the site team and fostered open dialogue between the site team and GTI team about any practical concerns. Facilitators played a critical role in preparing team members to set goals, select strategies, and evaluate efforts. Our previous work has shown a strong site team-facilitator bond likely contributed to success [43]. Building a team was central to the pilot’s success. As the team expanded from several to 28 members, redundancies in expertise and responsibilities emerged, such that loss of a single team member did not derail implementation efforts. To scale GTI, future work can study whether GTI-PrEP strategies can be successfully self-implemented without external facilitation.

To address PrEP prescribing disparities, GTI-PrEP was tailored to incorporate overall and race/ethnicity stratified data. Additionally, each GTI step was supplemented with literature on racial disparities in HIV prevention. Although this pilot was not powered to detect effectiveness, the observed numerical increase in PrEP prescribing to Black Veterans serves as an important proof of concept. These findings align with broader calls in the literature to move beyond merely describing health disparities and towards addressing them through implementation science [22, 23, 44–51].

Despite the novelty of our study question and approach, several limitations are present. First, this study was conducted in a single VA facility, limiting generalizability. While the pilot allowed for detailed process evaluation, it was not powered to assess effectiveness or the influence of site-level contextual characteristics. Second, given a lower performing site was selected, improvements may partially reflect regression to the mean rather than intervention effect. Third, data limitations include that PrEP prescribing remains relatively infrequent and there is no universally accepted threshold for defining a disparity. Despite these limitations, the study team worked closely with VA partners throughout the process to ensure consistency in implementation and measurement. Future research should test GTI-PrEP in a fully powered, cluster-randomized trial that includes assessments of the mechanisms of change at the provider and organizational levels.

## Conclusions

While there is a tension in implementation science between generalizability and contextualization, we have repeatedly demonstrated that GTI is a generalizable approach which can be contextualized to a variety of settings, including PrEP prescribing in the VA. This proof-of-concept pilot study leveraged national data, implementation strategy surveys, and high-performer qualitative interviews from across the VA to select and package implementation strategy bundles into the GTI-PrEP toolkit. This work offers a start toward using data to build sustainable implementation science methods to address health care disparities.

## Supplementary Information

Supplementary Material 1

Supplementary Material 2

## Data Availability

This quality improvement project was conducted as a non-research operations activity by the VA HIV, Hepatitis, and Related Conditions Programs (HHRC), with the stipulation that data would be presented in aggregate, given the sensitive information included in the dataset. However, interested parties can contact the Center for Health Equity Research and Promotion in the VA Pittsburgh Healthcare System (contact via Andrea.Krushinski@va.gov or shari.rogal@va.gov) for further inquiries or data requests.

## Abbreviations

CDC: Centers for Disease Control and Prevention
CFIR: Consolidated Framework for Implementation Research
ERIC: Expert Recommendations for Implementing Change
FY: Fiscal year
GI: Gastrointestinal
GTI: Getting to Implementation
GTO: Getting to Outcomes
HHRC: HIV, Hepatitis, and Related Conditions Program
HIV: Human immunodeficiency virus
ID: Infectious disease
LGBTQ +: Lesbian, Gay, Bisexual, Transgender, and Queer
PrEP: Pre-Exposure Prophylaxis
STI: Sexually transmitted infection
US: United States
VA: Veterans Health Administration

## References

[1] Centers for Disease Control and Prevention. Estimated HIV incidence and prevalence in the United States, 2018–2022. HIV Surveillance Supplemental Report. 2024; 29(1). https://www.cdc.gov/hiv-data/nhss/estimated-hiv-incidence-and-prevalence.html. Accessed August 16, 2024.

[2] US Preventive Services Task Force, Owens DK, Davidson KW, Krist AH, Barry MJ, Cabana M, et al. Preexposure Prophylaxis for the Prevention of HIV Infection: US Preventive Services Task Force Recommendation Statement. JAMA. 2019;321(22):2203–13. 10.1001/jama.2019.6390 31184747

[3] McCormack S, Dunn DT, Desai M, Dolling DI, Gafos M, Gilson R, et al. Pre-exposure prophylaxis to prevent the acquisition of HIV-1 infection (PROUD): effectiveness results from the pilot phase of a pragmatic open-label randomised trial. Lancet. 2016;387(10013):53–60. 26364263 10.1016/S0140-6736(15)00056-2 PMC4700047

[4] Baeten JM, Haberer JE, Liu AY, Sista N. Preexposure prophylaxis for HIV prevention: where have we been and where are we going? J Acquir Immune Defic Syndr. 2013;63 Suppl 2(0 2):S122-9. 23764623 10.1097/QAI.0b013e3182986f69 PMC3710117

[5] Morris ZS, Wooding S, Grant J. The answer is 17 years, what is the question: understanding time lags in translational research. J R Soc Med. 2011;104(12):510–20. 22179294 10.1258/jrsm.2011.110180 PMC3241518

[6] Rubin R. It Takes an Average of 17 Years for Evidence to Change Practice-the Burgeoning Field of Implementation Science Seeks to Speed Things Up. JAMA. 2023;329(16):1333–6. 37018006 10.1001/jama.2023.4387

[7] CDC issues guidelines urging the use of PrEP for HIV prevention. AIDS Policy Law. 2014;29(8):1-4. 25151679

[8] Centers for Disease Control and Prevention. Ending the HIV Epidemic in the US: Goals. https://www.cdc.gov/ehe/php/about/goals.html. Accessed 28 Apr 2025.

[9] Sell J, Chen R, Huber C, Parascando J, Nunez J. Primary Care Provider HIV PrEP Knowledge, Attitudes, and Prescribing Habits: A Cross-Sectional Survey of Late Adopters in Rural and Suburban Practice. J Prim Care Community Health. 2023;14. 10.1177/21501319221147254. 10.1177/21501319221147254 PMC9834790 36625276

[10] Jackson KJ, Chitle P, McCoy SI, White DAE. A Systematic Review of HIV Pre-exposure Prophylaxis (PrEP) Implementation in U.S. Emergency Departments: Patient Screening, Prescribing, and Linkage to Care. J Community Health. 2024;49(3):499–513. 38127296 10.1007/s10900-023-01320-7 PMC10981603

[11] Sullivan PS, DuBose SN, Castel AD, Hoover KW, Juhasz M, Guest JL, et al. Equity of PrEP uptake by race, ethnicity, sex and region in the United States in the first decade of PrEP: a population-based analysis. Lancet Reg Health Am. 2024;33:100738. 38659491 10.1016/j.lana.2024.100738 PMC11041841

[12] AIDSVu Releases New Data Highlighting Ongoing Inequities in PrEP Use among Black and Hispanic People and across Regions of the Country. 2023. https://aidsvu.org/news-updates/news-updates-aidsvu-releases-new-data-highlighting-ongoing-inequities-in-prep-use-among-black-and-hispanic-people-and-across-regions-of-the-county. Accessed September 13, 2024.

[13] CMS, CDC, & HRSA Launch HIV Health Improvement Affinity Group for State Medicaid Programs. 2016. https://www.hiv.gov/blog/cms-cdc-hrsa-launch-hiv-health-improvement-affinity-group-for-state-medicaid-programs. Accessed September 13, 2024.

[14] Waltz TJ, Powell BJ, Chinman MJ, Smith JL, Matthieu MM, Proctor EK, et al. Expert recommendations for implementing change (ERIC): Protocol for a mixed methods study. Implement Sci. 2014;9(1):1–12. 24669765 10.1186/1748-5908-9-39 PMC3987065

[15] Powell, Byron J, et al. Improving the implementation and sustainment of evidence-based practices in community mental health organizations: a study protocol for a matched-pair cluster randomized pilot study of the Collaborative Organizational Approach to Selecting and Tailoring Implementation Strategies (COAST-IS). Implementation science communications 1 (2020):1–13. 10.1186/s43058-020-00009-5 PMC7207049 32391524

[16] Powell BJ, Waltz TJ, Chinman MJ, Damschroder LJ, Smith JL, Matthieu MM, et al. A refined compilation of implementation strategies: results from the Expert Recommendations for Implementing Change (ERIC) project. Implement Sci. 2015;10:21. 25889199 10.1186/s13012-015-0209-1 PMC4328074

[17] Waltz TJ, Powell BJ, Matthieu MM, Damschroder LJ, Chinman MJ, Smith JL, et al. Use of concept mapping to characterize relationships among implementation strategies and assess their feasibility and importance: Results from the Expert Recommendations for Implementing Change (ERIC) study. Implement Sci. 2015;10(1):1–8. 26249843 10.1186/s13012-015-0295-0 PMC4527340

[18] Waltz TJ, Powell BJ, Fernandez ME, Abadie B, Damschroder LJ. Choosing implementation strategies to address contextual barriers: diversity in recommendations and future directions. Implement Sci. 2019;14(1):42. 31036028 10.1186/s13012-019-0892-4 PMC6489173

[19] Rogal SS, Yakovchenko V, Waltz TJ, Powell BJ, Kirchner JE, Proctor EK, et al. The association between implementation strategy use and the uptake of hepatitis C treatment in a national sample. Implement Sci. 2017;12(1):60. 28494811 10.1186/s13012-017-0588-6 PMC5425997

[20] Rogal SS, Yakovchenko V, Waltz TJ, Powell BJ, Gonzalez R, Park A, et al. Longitudinal assessment of the association between implementation strategy use and the uptake of hepatitis C treatment: Year 2. Implement Sci. 2019;14(1):36. 30961615 10.1186/s13012-019-0881-7 PMC6454775

[21] Rogal SS, Yakovchenko V, Morgan T, Bajaj JS, Gonzalez R, Park A, et al. Getting to implementation: a protocol for a Hybrid III stepped wedge cluster randomized evaluation of using data-driven implementation strategies to improve cirrhosis care for Veterans. Implement Sci. 2020;15(1):92. 33087156 10.1186/s13012-020-01050-7 PMC7579930

[22] Chinman M, Woodward EN, Curran GM, Hausmann LRM. Harnessing Implementation Science to Increase the Impact of Health Equity Research. Med Care. 2017;55 Suppl 9 Suppl 2:S16-S23. 10.1097/MLR.0000000000000769 PMC5639697 28806362

[23] McNulty M, Smith JD, Villamar J, Burnett-Zeigler I, Vermeer W, Benbow N, et al. Implementation Research Methodologies for Achieving Scientific Equity and Health Equity. Ethn Dis. 2019;29(Suppl 1):83–92. 30906154 10.18865/ed.29.S1.83 PMC6428169

[24] Powell BJ, Beidas RS, Lewis CC, Aarons GA, McMillen JC, Proctor EK, et al. Methods to Improve the Selection and Tailoring of Implementation Strategies. J Behav Health Serv Res. 2017;44(2):177–94. 26289563 10.1007/s11414-015-9475-6 PMC4761530

[25] Chinman M, McCarthy S, Hannah G, Byrne TH, Smelson DA. Using Getting To Outcomes to facilitate the use of an evidence-based practice in VA homeless programs: a cluster-randomized trial of an implementation support strategy. Implement Sci. 2017;12(1):34. 28279207 10.1186/s13012-017-0565-0 PMC5345223

[26] Chinman M, Acosta J, Ebener P, Malone PS, Slaughter ME. A Cluster-Randomized Trial of Getting To Outcomes’ Impact on Sexual Health Outcomes in Community-Based Settings. Prev Sci. 2018;19(4):437–48. 28971273 10.1007/s11121-017-0845-6 PMC5880746

[27] Smith JL, Ritchie MJ, Kim B, Miller CJ, Chinman MJ, Adam Kelly P, et al. Getting to Fidelity: Consensus Development Process to Identify Core Activities of Implementation Facilitation. Glob Implement Res Appl. 2024;4(2):151–66. 10.1007/s43477-024-00119-5 PMC11100021 38765294

[28] Yakovchenko V, Rogal SS, Goodrich DE, Lamorte C, Neely B, Merante M, et al. Getting to implementation: Adaptation of an implementation playbook. Front Public Health. 2022;10:980958. 36684876 10.3389/fpubh.2022.980958 PMC9853037

[29] Department of Veterans Affairs Office of Research & Development. Program Guide 1200.21: VHA Operations Activities That May Constitute Research. 2019. https://www.research.va.gov/resources/policies/ProgramGuide-1200-21-VHA-Operations-Activities.pdf. Accessed April 29, 2025.

[30] Proctor EK, Powell BJ, McMillen JC. Implementation strategies: recommendations for specifying and reporting. Implement Sci. 2013;8:139. 24289295 10.1186/1748-5908-8-139 PMC3882890

[31] Chinman M, Early D, Ebener P, Hunter S, Imm P, Jenkins P, et al. Getting To Outcomes: a community-based participatory approach to preventive interventions. J Interprof Care. 2004;18(4):441–3. 15801559 10.1080/13561820400011727

[32] Chinman M, Imm P, Wandersman A. Getting to Outcomes 2004: Promoting Accountability Through Methods and Tools for Planning, Implementation, and Evaluation. Santa Monica, CA: RAND Corporation; 2004. Contract No.: TR-101.

[33] Ending the HIV Epidemic Priority Jurisdictions. https://www.hiv.gov/federal-response/ending-the-hiv-epidemic/jurisdictions. Accessed 29 April 2025.

[34] Kim B, Miller CJ, Ritchie MJ, Smith JL, Kirchner JE, Stolzmann K, et al. Time-motion analysis of external facilitation for implementing the Collaborative Chronic Care Model in general mental health clinics: Use of an interval-based data collection approach. Implement Res Pract. 2022;3:26334895221086276. 37091094 10.1177/26334895221086275 PMC9924237

[35] van Grootel L, van Wesel F, O’Mara-Eves A, Thomas J, Hox J, Boeije H. Using the realist perspective to link theory from qualitative evidence synthesis to quantitative studies: Broadening the matrix approach. Res Synth Methods. 2017;8(3):303–11. 28429447 10.1002/jrsm.1241

[36] Miller CJ, Barnett ML, Baumann AA, Gutner CA, Wiltsey-Stirman S. The FRAME-IS: a framework for documenting modifications to implementation strategies in healthcare. Implement Sci. 2021;16(1):36. 33827716 10.1186/s13012-021-01105-3 PMC8024675

[37] Damschroder LJ, Reardon CM, Widerquist MAO, Lowery J. The updated Consolidated Framework for Implementation Research based on user feedback. Implement Sci. 2022;17(1):75. 36309746 10.1186/s13012-022-01245-0 PMC9617234

[38] Proctor EK, Powell BJ, Feely M. Measurement in dissemination and implementation science. In: Beidas RSKP, editor. Dissemination and implementation of evidence-based practices in child and adolescent mental health. New York: Oxford University Press; 2014. p. 22–43.

[39] Powell BJ, Waltz TJ, Chinman MJ, Damschroder LJ, Smith JL, Matthieu MM, et al. A refined compilation of implementation strategies: Results from the Expert Recommendations for Implementing Change (ERIC) project. Implement Sci. 2015. 10.1186/s13012-015-0209-1 PMC4328074 25889199

[40] Szymczak JE, Petty LA, Gandhi TN, Neetz RA, Hersh A, Presson AP, et al. Protocol for a parallel cluster randomized trial of a participatory tailored approach to reduce overuse of antibiotics at hospital discharge: the ROAD home trial. Implement Sci. 2024;19(1):23. 38439076 10.1186/s13012-024-01348-w PMC10910678

[41] Cook CR, Lyon AR, Locke J, Waltz T, Powell BJ. Adapting a Compilation of Implementation Strategies to Advance School-Based Implementation Research and Practice. Prev Sci. 2019;20(6):914–35. 31152328 10.1007/s11121-019-01017-1 PMC8943907

[42] Kerbler MK, Isaacs C, Eatmon C, Reid J, Davis KW. Impact of an HIV pre-exposure prophylaxis dashboard on veteran PrEP enrollment. J Am Pharm Assoc (2003). 2024;64(2):471–5. 38215824 10.1016/j.japh.2024.01.002

[43] Yakovchenko V, Merante M, Chinman MJ, Neely B, Lamorte C, Gibson S, et al. The “good enough” facilitator: elucidating the role of working alliance in the mechanism of facilitation. Implement Sci Commun. 2025;6(1):22. 40001234 10.1186/s43058-025-00705-0 PMC11863522

[44] Bradley CD, Irie WC, Geng EH. Situating implementation science (IS) in res(IS)tance: a conceptual frame toward the integration of scholarship from the black radical tradition. Front Public Health. 2023;11:1286156. 38274530 10.3389/fpubh.2023.1286156 PMC10808293

[45] Shelton RC, Adsul P, Oh A, Moise N, Griffith DM. Application of an antiracism lens in the field of implementation science (IS): Recommendations for reframing implementation research with a focus on justice and racial equity. Implement Res Pract. 2021;2:26334895211049480. 37089985 10.1177/26334895211049482 PMC9978668

[46] Purnell TS, Calhoun EA, Golden SH, Halladay JR, Krok-Schoen JL, Appelhans BM, et al. Achieving Health Equity: Closing The Gaps In Health Care Disparities, Interventions. And Research Health Aff (Millwood). 2016;35(8):1410–5. 27503965 10.1377/hlthaff.2016.0158

[47] Woodward EN, Matthieu MM, Uchendu US, Rogal S, Kirchner JE. The health equity implementation framework: proposal and preliminary study of hepatitis C virus treatment. Implement Sci. 2019;14(1):26. 30866982 10.1186/s13012-019-0861-y PMC6417278

[48] Baumann AA, Cabassa LJ. Reframing implementation science to address inequities in healthcare delivery. BMC Health Serv Res. 2020;20(1):190. 32164706 10.1186/s12913-020-4975-3 PMC7069050

[49] Galaviz KI, Breland JY, Sanders M, Breathett K, Cerezo A, Gil O, et al. Implementation Science to Address Health Disparities During the Coronavirus Pandemic. Health Equity. 2020;4(1):463–7. 33111032 10.1089/heq.2020.0044 PMC7585610

[50] Brownson RC, Kumanyika SK, Kreuter MW, Haire-Joshu D. Implementation science should give higher priority to health equity. Implement Sci. 2021;16(1):28. 33740999 10.1186/s13012-021-01097-0 PMC7977499

[51] Woodward EN, Singh RS, Ndebele-Ngwenya P, Melgar Castillo A, Dickson KS, Kirchner JE. A more practical guide to incorporating health equity domains in implementation determinant frameworks. Implement Sci Commun. 2021;2(1):61. 34090524 10.1186/s43058-021-00146-5 PMC8178842

