# Supplementary material for: Getting to Implementation for HIV Pre-Exposure Prophylaxis (GTI-PrEP): A data-driven approach to PrEP prescribing
Source: Implement Sci Commun. 2025 Jun 2;6:71. doi: 10.1186/s43058-025-00749-2 (PMC12131467; doi:10.1186/s43058-025-00749-2)
Supplement: Supplementary file 1 — Supplementary Material 1 [file 43058_2025_749_MOESM1_ESM.docx]

**Revised Standards for Quality Improvement Reporting Excellence (SQUIRE 2.0)
September 15, 2015**

| **Text Section and Item Name** | **Page #** | **Section or Item Description** |
| --- | --- | --- |
| **Notes to authors** |  | - The SQUIRE guidelines provide a framework for reporting new   knowledge about how to improve healthcare   - The SQUIRE guidelines are intended for reports that describe [system](#_bookmark13) level work to improve the quality, safety, and value of healthcare, and used methods to establish that observed outcomes were due to the [intervention(s).](#_bookmark8) - A range of approaches exists for improving healthcare. SQUIRE may be adapted for reporting any of these. - Authors should consider every SQUIRE item, but it may be inappropriate or unnecessary to include every SQUIRE element in a particular manuscript. - The SQUIRE Glossary contains definitions of many of the key words in SQUIRE. - The Explanation and Elaboration document provides specific examples of well-written SQUIRE items, and an in-depth explanation of each item. - Please cite SQUIRE when it is used to write a manuscript. |
| **Title and Abstract** |  |  |
| **1. Title** | 1 | Indicate that the manuscript concerns an [initiative](#_bookmark6) to improve healthcare (broadly defined to include the quality, safety, effectiveness, patient- centeredness, timeliness, cost, efficiency, and equity of healthcare) |
| **2. Abstract** | 3-4 | 1. Provide adequate information to aid in searching and indexing 2. Summarize all key information from various sections of the text using the abstract format of the intended publication or a structured summary such as: background, local [problem,](#_bookmark10) methods, interventions, results, conclusions |
| **Introduction** |  | *Why did you start?* |
| [**3. Problem**](#_bookmark10) [**Description**](#_bookmark10) | 5-6 | Nature and significance of the local [problem](#_bookmark10) |
| **4. Available knowledge** | 5-7 | Summary of what is currently known about the [problem,](#_bookmark10) including relevant previous studies |
| **5.** [**Rationale**](#_bookmark12) | 6-7 | Informal or formal frameworks, models, concepts, and/or [theories](#_bookmark14) used to explain the [problem,](#_bookmark10) any reasons or [assumptions](#_bookmark0) that were used to develop the [intervention(s),](#_bookmark8) and reasons why the [intervention(s)](#_bookmark8) was  expected to work |
| **6. Specific aims** | 7 | Purpose of the project and of this report |
| **Methods** |  | *What did you do?* |
| **7.** [**Context**](#_bookmark1) | 7-10 | Contextual elements considered important at the outset of introducing the [intervention(s)](#_bookmark8) |
| **8.** [**Intervention(s)**](#_bookmark8) | 10-12 | 1. Description of the [intervention(s)](#_bookmark8) in sufficient detail that others could reproduce it 2. Specifics of the team involved in the work |
| **9. Study of the Intervention(s)** | 11-12 | 1. Approach chosen for assessing the impact of the [intervention(s)](#_bookmark8) 2. Approach used to establish whether the observed outcomes were due to the [intervention(s)](#_bookmark8) |
| **10. Measures** | 8-10, 11-12 | 1. Measures chosen for studying [processes](#_bookmark11) and outcomes of the [intervention(s),](#_bookmark8) including rationale for choosing them, their operational definitions, and their validity and reliability 2. Description of the approach to the ongoing assessment of contextual elements that contributed to the success, failure, efficiency, and cost 3. Methods employed for assessing completeness and accuracy of data |
| **11. Analysis** | 9, 11-12 | 1. Qualitative and quantitative methods used to draw [inferences](#_bookmark5) from the data 2. Methods for understanding variation within the data, including the   effects of time as a variable |
| **12. Ethical Considerations** | 7-8 | [Ethical aspects](#_bookmark2) of implementing and studying the [intervention(s)](#_bookmark8) and how they were addressed, including, but not limited to, formal ethics review and potential conflict(s) of interest |
| **Results** |  | *What did you find?* |
| **13. Results** | 12-18 | 1. Initial steps of the [intervention(s)](#_bookmark8) and their evolution over time (*e.g.*, time-line diagram, flow chart, or table), including modifications made to the intervention during the project 2. Details of the [process](#_bookmark11) measures and outcome 3. Contextual elements that interacted with the [intervention(s)](#_bookmark8) 4. Observed associations between outcomes, interventions, and relevant contextual elements 5. Unintended consequences such as unexpected benefits, problems, failures, or costs associated with the [intervention(s).](#_bookmark8) 6. Details about missing data |
| **Discussion** |  | *What does it mean?* |
| **14. Summary** | 18 | 1. Key findings, including relevance to the [rationale](#_bookmark12) and specific aims 2. Particular strengths of the project |
| **15. Interpretation** | 18-21 | 1. Nature of the association between the [intervention(s)](#_bookmark8) and the outcomes 2. Comparison of results with findings from other publications 3. Impact of the project on people and [systems](#_bookmark13) 4. Reasons for any differences between observed and anticipated outcomes, including the influence of [context](#_bookmark1) 5. Costs and strategic trade-offs, including [opportunity costs](#_bookmark9) |
| **16. Limitations** | 21-22 | 1. Limits to the [generalizability](#_bookmark3) of the work 2. Factors that might have limited [internal validity](#_bookmark7) such as confounding, bias, or imprecision in the design, methods, measurement, or analysis 3. Efforts made to minimize and adjust for limitations |
| **17. Conclusions** | 22 | 1. Usefulness of the work 2. Sustainability 3. Potential for spread to other [contexts](#_bookmark1) 4. Implications for practice and for further study in the field 5. Suggested next steps |
| **Other information** |  |  |
| **18. Funding** | 23 | Sources of funding that supported this work. Role, if any, of the funding organization in the design, implementation, interpretation, and reporting |

**Table 2. Glossary of key terms used in SQUIRE 2.0. This Glossary provides the intended meaning of selected words and phrases as they are used in the SQUIRE 2.0 Guidelines. They may, and often do, have different meanings in other disciplines, situations, and settings .**

**Assumptions**

Reasons for choosing the activities and tools used to bring about changes in healthcare services at the [system](#_bookmark13) level.

# Context

Physical and sociocultural makeup of the local environment (for example, external environmental factors, organizational dynamics, collaboration, resources, leadership, and the like), and the interpretation of these factors (“sense-making”) by the healthcare delivery professionals, patients, and caregivers that can affect the effectiveness and [generalizability](#_bookmark3) of [intervention(s).](#_bookmark8)

# Ethical aspects

The value of [system-](#_bookmark13)level [initiatives](#_bookmark6) relative to their potential for harm, burden, and cost to the stakeholders. Potential harms particularly associated with efforts to improve the quality, safety, and value of healthcare services include [opportunity costs,](#_bookmark9) invasion of privacy, and staff distress resulting from disclosure of poor performance.

# Generalizability

The likelihood that the [intervention(s)](#_bookmark8) in a particular report would produce similar results in other settings, situations, or environments (also referred to as external validity).

# Healthcare improvement

Any systematic effort intended to raise the quality, safety, and value of healthcare services, usually done at the [system](#_bookmark13) level. We encourage the use of this phrase rather than “quality improvement,” which often refers to more narrowly defined approaches.

# Inferences

The meaning of findings or data, as interpreted by the stakeholders in healthcare services – improvers, healthcare delivery professionals, and/or patients and families

# Initiative

A broad term that can refer to organization-wide programs, narrowly focused projects, or the details of specific interventions (for example, planning, execution, and assessment)

# Internal validity

Demonstrable, credible evidence for efficacy (meaningful impact or change) resulting from introduction of a specific intervention into a particular healthcare [system.](#_bookmark13)

# Intervention(s)

The specific activities and tools introduced into a healthcare [system](#_bookmark13) with the aim of changing its performance for the better. Complete description of an intervention includes its inputs, internal activities, and outputs (in the form of a logic model, for example), and the mechanism(s) by which these components are expected to produce changes in a [system’s](#_bookmark13) performance.

# Opportunity costs

Loss of the ability to perform other tasks or meet other responsibilities resulting from the diversion of resources needed to introduce, test, or sustain a particular [improvement](#_bookmark4) initiative

# Problem

Meaningful disruption, failure, inadequacy, distress, confusion or other dysfunction in a healthcare service delivery [system](#_bookmark13) that adversely affects patients, staff, or the [system](#_bookmark13) as a whole, or that prevents care from reaching its full potential

# Process

The routines and other activities through which healthcare services are delivered

# Rationale

Explanation of why particular [intervention(s)](#_bookmark8) were chosen and why it was expected to work, be sustainable, and be replicable elsewhere.

# Systems

The interrelated structures, people, [processes,](#_bookmark11) and activities that together create healthcare services for and with individual patients and populations. For example, systems exist from the personal self- care system of a patient, to the individual provider-patient dyad system, to the microsystem, to the macrosystem, and all the way to the market/social/insurance system. These levels are nested within each other.

# Theory or theories

Any “reason-giving” account that asserts causal relationships between variables (causal theory) or that makes sense of an otherwise obscure [process](#_bookmark11) or situation (explanatory theory). Theories come in many forms, and serve different purposes in the phases of [improvement](#_bookmark4) work. It is important to be explicit and well-founded about any informal and formal theory (or theories) that are used.
