# Supplementary material for: Getting to Implementation for HIV Pre-Exposure Prophylaxis (GTI-PrEP): A data-driven approach to PrEP prescribing
Source: Implement Sci Commun. 2025 Jun 2;6:71. doi: 10.1186/s43058-025-00749-2 (PMC12131467; doi:10.1186/s43058-025-00749-2)
Supplement: Supplementary file 2 — Supplementary Material 2 [file 43058_2025_749_MOESM2_ESM.docx]

**Supplemental Table 1. CFIR Items change in post GTI survey**

| **Items ranked less of a barrier in post survey** |
| --- |
| PrEP improvement is presented in an accessible way. |
| Clinicians network with colleagues outside of our station about HIV prevention and care. |
| Clinicians encourage each other to provide PrEP. |
| Our station is generally supportive of innovation. |
| Clinicians have the time they need to do PrEP. |
| Clinicians have access to the information that is needed to do PrEP. |
| Clinicians at my station feel burned out from their work. |
| We have engaged with Veterans to understand their perspectives on PrEP. |
| We have a plan to evaluate how well our PrEP improvement efforts are working. |
| **Items ranked more of a barrier in post survey** |
| Clinicians believe the evidence behind PrEP is strong. |
| Clinicians believe doing PrEP will benefit Veterans more than current practices. |
| Compared to other efforts, PrEP is a high priority. |
| Clinicians feel safe to try new methods to improve care. |
| Clinicians are confident in their ability to complete tasks related to PrEP. |
| Clinicians are committed to PrEP improvement. |
| Administrative and scheduling staff will support PrEP. |
| **No change** |
| Leadership mandated PrEP without considering local staff. |
| We would be able to change how PrEP is done to fit in our station. |
| We would be able to pilot PrEP as to start small and change our approach if needed. |
| Clinicians believe PrEP is simple to implement. |
| PrEP is cost-effective. |
| Clinicians believe that the PrEP addresses the needs of Veterans. |
| Veterans have the resources they need to do PrEP. |
| Policies and incentives support clinicians in providing PrEP. |
| We have low staff turnover in our station. |
| Communication works well in our station. (Communications could involve clinicians, administrative staff, leadership, and other staff members across departments.) |
| Clinicians are receptive to implementing PrEP. |
| Clinicians believe that PrEP fits well into their current workflow. |
| Clinicians will receive positive reinforcement from leadership for improving PrEP. |
| Goals and performance data are clearly communicated to staff. |
| Leadership cares about improving PrEP. |
| We have the space and equipment (infrastructure) that we need for PrEP. |
| We have the right staff for PrEP. |
| Veterans will agree to PrEP. |
| Clinicians have thought about and planned for how to improve PrEP. |
| We know the right people to involve in our PrEP improvement. |
| There are supportive people outside of our station who will help our PrEP efforts. |
| Clinicians will be able to consistently deliver PrEP. |
